# Molecular switches of the *κ* opioid receptor triggered by 6′-GNTI and 5′-GNTI

**DOI:** 10.1038/srep18913

**Published:** 2016-01-08

**Authors:** Jianxin Cheng, Xianqiang Sun, Weihua Li, Guixia Liu, Yaoquan Tu, Yun Tang

**Affiliations:** 1 Shanghai Key Laboratory of New Drug Design, School of Pharmacy, East China University of Science and Technology, Shanghai 200237, China; 2 Division of Theoretical Chemistry and Biology, School of Biotechnology, KTH Royal Institute of Technology, S-106 91 Stockholm, Sweden

## Abstract

The *κ* opioid receptor (*κ*OR) is a member of G-protein-coupled receptors, and is considered as a promising drug target for treating neurological diseases. *κ*OR selective 6′-GNTI was proved to be a G-protein biased agonist, whereas 5′-GNTI acts as an antagonist. To investigate the molecular mechanism of how these two ligands induce different behaviors of the receptor, we built two systems containing the 5′-GNTI-*κ*OR complex and the 6′-GNTI-*κ*OR complex, respectively, and performed molecular dynamics simulations of the two systems. We observe that transmembrane (TM) helix 6 of the *κ*OR rotates about 4.6^o^ on average in the *κ*OR-6′-GNTI complex. Detailed analyses of the simulation results indicate that E297^6.58^ and I294^6.55^ play crucial roles in the rotation of TM6. In the simulation of the *κ*OR-5′-GNTI system, it is revealed that 5′-GNTI can stabilize TM6 in the inactive state form. In addition, the kink of TM7 is stabilized by a hydrogen bond between S324^7.47^ and the residue V69^1.42^ on TM1.

Opioid receptors (ORs) are important members of G-protein-coupled receptors (GPCRs) and the main targets for analgesics^1^,^2^,^3^. There are three subtypes of ORs, namely - the *μ*OR, *δ*OR and *κ*OR. Among the three subtypes, the *κ*OR is a promising drug target for alleviating pain with a possible lower abuse potential^4^,^5^,^6^. As a result, the *κ*OR subtype represents an excellent target to treat neurological disorders^7^,^8^,^9^,^10^,^11^,^12^,^13^. Selective agonists targeting the *κ*OR have been developed to treat related disorders^14^, and antagonists selectively targeting the *κ*OR have proved to be effective on curing depressant and anxiolytic diseases^15^,^16^,^17^,^18^.

Among all the known ligands targeting the *κ*OR, 5′-GNTI (5′-Guanidinonaltrindole) and 6′-GNTI (6′-Guanidinonaltrindole) are an interesting pair (Fig. 1). These two compounds are derivatives of Naltrindole (NTI), a highly potent *δ*OR-selective antagonist^19^,^20^. The difference between 5′-GNTI and 6′-GNTI is only in the substitution site of the guanidinium group. However, these two compounds lead to distinct behaviors of the *κ*OR. 5′-GNTI works as an antagonist to block or dampen the activation of the *κ*OR^21^,^22^, whereas 6′-GNTI has proved to be a G-protein biased agonist to trigger the activation of the receptor^23^,^24^. It has been verified that the guanidinium group in 5′-GNTI can interact with the residue E297^6.58^ (Ballesteros/Weinstein numbering^25^) on transmembrane helix (TM) 6^20^. It is interesting that the guanidinium group of 6′-GNTI has also been suggested to be interacting with E297^6.58^. Upon the binding of 6′-GNTI to the *κ*OR, a rotation of TM6 of the receptor is likely triggered which leads to the subsequent activation behaviors of the receptor. Unfortunately, all these interaction patterns and the related activation mechanisms were surmised mostly based on homology models, which largely limit our understanding on the behaviors of the *κ*OR.

Thanks to Stevens and his co-workers, the crystal structure of the human *κ*OR in complex with its selective antagonist JDTic^26^ and the crystal structures of the *δ*OR were reported recently^27^,^28^. These structures provide us atomic structural information on opioid receptors^29^,^30^. Unfortunately, all these crystal structures were obtained with the receptors in the inactive states. Furthermore, for new analgesics design, it is indispensable for us to exhaustively grasp the opioid activation mechanisms^31^. Therefore, to further study the behaviors of the *κ*OR induced by different kinds of ligands, large amount of work still needs to be done.

To explicate the activation mechanism of the *κ*OR triggered by 6′-GNTI, as well as the interactions between the antagonist 5′-GNTI and the *κ*OR, we carried out sub-microsecond unbiased molecular dynamics (MD) simulations of systems with the *κ*OR in the apo state and with 5′-GNTI-*κ*OR and 6′-GNTI-*κ*OR complexes, respectively, to provide active and inactive models of the GPCR based on the action of the so-called “molecular switches“^32^,^33^ buried in the receptor. TM6 of the *κ*OR was observed to rotate about 4.6^o^ on average in the *κ*OR-6′-GNTI complex. Helices TM7 and TM2 highly responded to the activation signal in 6′-GNTI systems too. In the simulation of the *κ*OR-5′-GNTI system, 5′-GNTI could stabilize TM6 in the inactive state form.

## Results

### Initial interaction modes obtained from molecular docking

**Figure 2:**
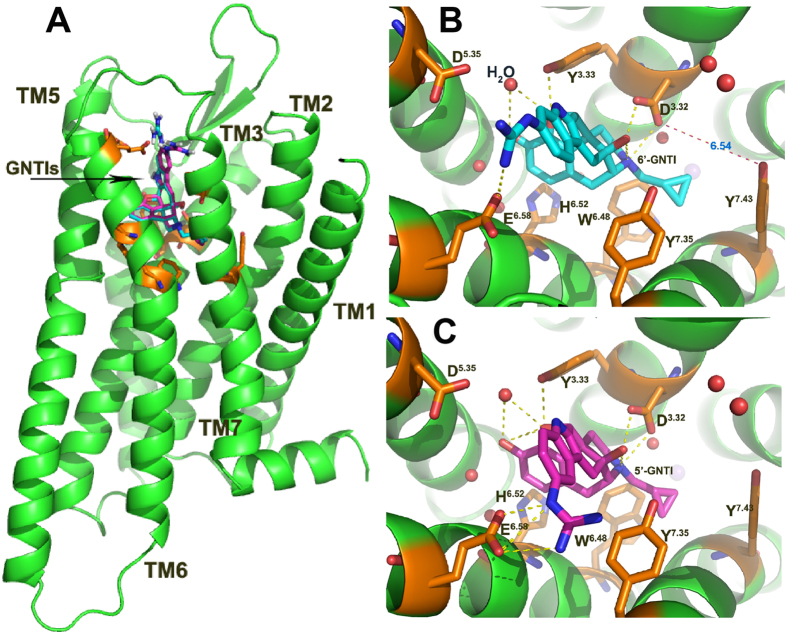
Docking poses. Alignment of the docking poses (**A**) and key residues (**B/C**) in the GNTI-*κ*OR complexes. The backbones of the ligands were in green (6′-GNTI) and pink (5′-GNTI). The hydrogen bond interactions were shown by yellow dot lines.

The docking poses of 6′-GNTI and 5′-GNTI are almost identical with each other as shown in Fig. 2. Strong electrostatic interaction is formed between the protonated nitrogen on the heterocyclic ring and D138^3.32^ in each complex. The phenolic hydroxyl group of both ligands interacts with H291^6.52^ bridged by two water molecules and the cyclopropyl group interacts with W287^6.48^. Meanwhile, Y139^3.33^ and Y320^7.43^ also contribute to the stabilization of the two ligands at the orthosteric site. Because of the difference in the substitution site of the guanidinium group in 6′-GNTI and 5′-GNTI, different interaction patens were observed when the two ligands bind to the *κ*OR. The guanidinium group of 5′-GNTI is located in the cavity formed by the residues on TM6 and TM7 and forms three hydrogen bonds with E297^6.58^, whereas the guanidinium group of 6′-GNTI is positioned in a cavity formed by the residues on TM5 and TM6 and forms only one hydrogen bond with the residue E297^6.58^. In addition, the docking poses of the two GNTIs are almost identical with the conformation of NTI adopted in the crystal structure of the NTI-*δ*OR complex. With the structures of 5′-GNTI or 6′-GNTI in the orthosteric pocket of the *κ*OR and the *κ*OR in the apo state, we built three systems, 5′-GNTI-*κ*OR, 6′-GNTI-*κ*OR, and the *κ*OR in the apo state, to be used for the following simulations.

### Overview of the MD simulation results

**Figure 3:**
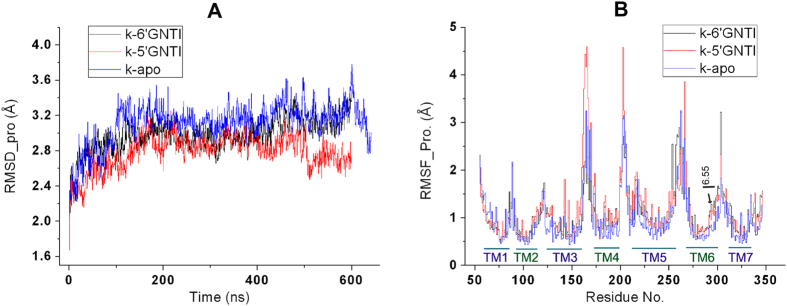
Overview of the MD simulations. (**A**) RMSD values of the protein in three 600 ns systems. (**B**) RMSF values for all the residues in the three systems calculated from 550 ns to 600 ns.

In our simulations, the systems reached the equilibrium at about 100 ns as indicated from the root mean square deviation (RMSD) values in the simulations (Fig. 3A). The RMSD values of the *κ*OR with respect to its crystal structure range from 2.0 to 3.6 Å (Fig. 3A). The ligands undergo comparably small conformational changes as indicated by their RMSD values, which range from 0.2 to 1.2 Å (Fig. S1). In addition, we also calculated the root mean square fluctuations (RMSFs) of the systems to analyze the fluctuations of the receptor. The loop section of the receptor exhibits a much larger fluctuation as compared to the conserved TM section (Fig. 3B). IL (intracellular loop) 2, EL (extracellular loop) 2 and IL3, show significant fluctuations, which are related to the fact that there are many residues on these three loops. The large fluctuation of the loop section reflects the significant conformational changes of the residues adjacent to these loops.

Ligand cluster analysis was performed on the two 600 ns GNTIs-*κ*OR systems, and two sub-state conformations were obtained under a 0.2 Å RMSD cut-off (Table 1). The main diversity between the two clusters was the tilt direction of indole ring of GNTIs (Fig. S2). One direction was outward if viewed from the extracellular side, whereas the other one was inward. Interestingly, the tilt direction of indole ring was inward among Cluster 1 of 6′-GNTI-*κ*OR system, while that was outward in 600 ns 5′-GNTI-*κ*OR system. Meanwhile, the population of Cluster 1 ran up to a half in 6′-GNTI-*κ*OR system, whereas that was in the majority, amount to 81.4% in 600 ns 5′-GNTI-*κ*OR complexes. Given the corresponding changes of guanidinium group (Fig. S2) and their important roles in GNTIs binding, we think the above-mentioned differences were mainly due to the diverse performances of guanidinium group between the two GNTIs-*κ*OR systems.

### Movements of the transmembrane helices

Comparisons of GPCR crystal structures in the inactive and active states have revealed several conserved switches to explain the inhibition or activation mechanisms of GPCRs^34^. One of the conserved switches is the large-scale rearrangements of the TM helices, including the rotation of TM6, the movement of TM5, the slight rotation and upward movement of TM3, and the inward movements of TM7 and TM1^35^. TM6 and TM3 are at the heart of any common activation pathways, because they are coupled with critical conserved residues and are in direct contact with other helices except for TM1^36^. We thus analyzed the conformational changes of TM6 and TM3.

**Figure 4:**
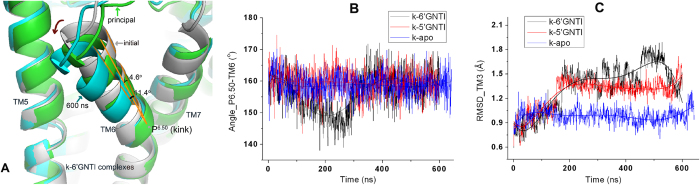
The motions of TM6 and TM3. (**A**) The motion of TM6 at the extracellular side in the 6′-GNTI-*κ*OR complex during the molecular dynamics simulations. (**B**) Evolution of the kink angle of TM6 in the simulations. (**C**) RMSDs of TM3 in the three 600 ns systems.

A sharp kink of TM6 has been observed in the crystal structure of the *κ*OR as well as in other family A GPCR structures with P^6.50^ as the pivot point^36^,^37^. Such a kink is preserved in our MD simulations (Fig. 4B). However, the kink angle of TM6 with P289^6.50^ as the pivot point varies with the simulation time. It changes from 160^o^ to 140 ^o^ at the initial stage of the simulation and then increases to about 159^o^ again (Fig. 4B and Table S1). Besides the kink of TM6, TM6 in the 6′-GNTI-*κ*OR complex counterclockwise rotates about 4.6^o^ on average, even 11.4^o^ at 600 ns conformation viewed from the extracellular side of the receptor (Fig. 4A). This observation is consistent with the published results^35^. In contrast, the TM6 kink in the 5′-GNTI-*κ*OR complex or the apo *κ*OR form was found to be stabilized at about 160^o^ and the counterclockwise rotation of TM6 was not observed from our simulations. We thus believe that the variation of the kink angle and the counterclockwise rotation of TM6 can be used to explain the different behaviors of the *κ*OR triggered by 5′-GNTI and 6′-GNTI.

**Figure 5:**
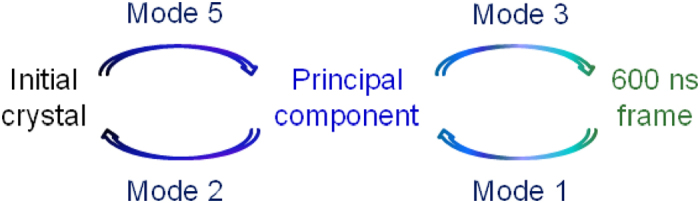
NMA profiles of TM6 conformational changes of the initial crystal structure, principal component and final 600 ns state (Fig. 4A) in 6′-GNTI-*κ*OR system. The arrows mean the directions of these conformational changes.

Normal mode analysis (NMA)^38^ is an efficient method for predicting inherent flexibilities in biological macromolecules. We performed NMA on the typical structures of the initial crystal structure, principal component and final 600 ns state in Fig. 4A, to detect the intrinsic motions of *κ*OR. The low-frequency modes of *κ*OR produced by the NMA reflected the global motions of the receptor and were often related to biological functions^39^. The first two lowest-frequency motion modes (modes 1 and 2) on the final 600 ns state and principal component were relevant to the transition from active-like to inactive (Fig. 5). Interestingly, NMA of the initial crystal structure revealed that the inactive state had an intrinsic potential to change back to the active-like conformation. However, the modes relevant to this transition were only among the fifth and third lowest-frequency motion modes. Therefore, unless an external force or ligand was present, *κ*OR would favour the inactive state within the circuit. The consistency between the results of MD and NMA supports the efficiency of both methods in studying the large-scale motions of TM6 domains.

The conformational changes of TM3 and TM7 in the simulations can also be used to disclose the role of 5′-GNTI and 6′-GNTI in the activation of the *κ*OR. The binding of GNTIs causes large conformational change of TM3 in the GNTIs−*κ*OR complexes as shown in our simulations. In the simulation of the 5′-GNTI-*κ*OR complex, we observed a clockwise rotation of TM7 if viewed from the extracellular side (Fig. S4A). We attribute the clockwise rotation of TM7 to the fluctuation of ECL3 (Video S1A). ECL3 is mainly composed of hydrophilic residues, such as Ser or His. These residues form strong interactions with the guanidino group of 5′-GNTI, which is responsible for the clockwise rotation of TM7.

### Comparison of the binding interactions of GNTIs with the κOR

Hydrogen bonds between the residues D138^3.32^, Y139^3.33^, H291^6.52^, and E297^6.58^ on the receptor and the ligands are formed for most of the time in the simulations of the 5′-GNTI-*κ*OR and 6′-GNTI-*κ*OR complexes (Table 2 and Fig. 2). E297^6.58^ forms two HBs with the ligands and contributes significantly to the stabilization of the ligands at the orthosteric site. In addition, the protonated nitrogen in either 6′-GNTI or 5′-GNTI is not able to form any HBs with D138^3.32^ (Table 2). This can be considered as a common binding mechanism of opioid towards ORs^40^.

### Contribution of I294^6.55^ to the conformational change of TM6

**Figure 6:**
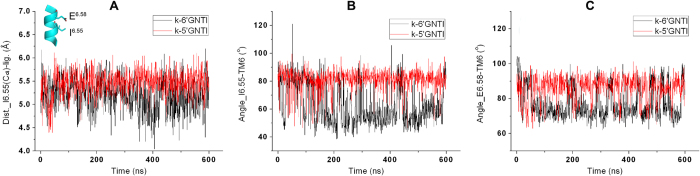
Comparisons of the contribution of I294^6.55^. (**A**) Evolution of the minimum distances between C_α_ of the residue I294^6.55^ and GNTIs. (**B,C**) Evolution of the angle between the residue I294^6.55^ and TM6 (**B**) or between the residue E297^6.58^ and TM6 (**C**).

Detailed analysis of the trajectory from the simulation of the 5′-GNTI-*κ*OR system showed that the minimum distance between C_α_ of I294^6.55^ and 5′-GNTI is mainly stabilized at about 5.5 Å (Fig. 6 and Table S1). The corresponding distance in the 6′-GNTI-*κ*OR complex fluctuates between 4 and 6 Å, with the average at about 5.2 Å. The average minimum distance between C_α_ of I294^6.55^ and 6′-GNTI is about 0.3 Å shorter than that between C_α_ of I294^6.55^ and 5′-GNTI (Fig. 6A and Video S1B). The shorter distance between C_α_ of I294^6.55^ and 6′-GNTI can raise the steric clash between 6′-GNTI and I294^6.55^. The existence of the steric clash between I294^6.55^ and 6′-GNTI can be further indicated from the temporal evolutions of an angle between residue I294^6.55^ and TM6 (Fig. 6B). Because of the steric clash, this angle undergoes a significant fluctuation between 40^o^ and 120^o^, with an average value at about 63^o^. On the contrary, the corresponding angle is stabilized at about 81^o^ in the 5′-GNTI-*κ*OR complex. The RMSF values also reflect that the fluctuation of the angle in the 6′-GNTI-*κ*OR complex is larger than in the 5′-GNTI-*κ*OR complex (Fig. 3B). In the simulation of the *κ*OR in the apo state, this angle fluctuated between 40^o^ and 100^o^ freely (Fig. S5A).

The strong steric clash between I294^6.55^ and 6′-GNTI has a significant influence on the conformational states of E297^6.58^ (Fig. 6C). The angles between residue E297^6.58^ and TM6 is mainly stabilized at about 77^o^ in the 6′-GNTI-*κ*OR complex, whereas in the 5′-GNTI-*κ*OR complex it is at about 86^o^ on average. The large influence could also be reflected from the diverse performances of ligand cluster among 6′-GNTI-*κ*OR interaction (Table 1 and Fig. S2).

### Conformational change of TM7

**Figure 7:**
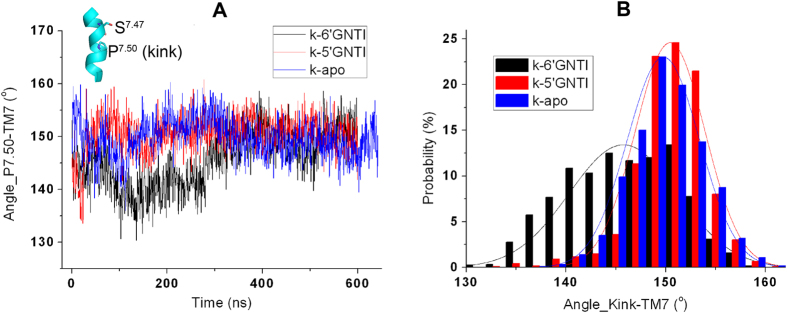
The motions of TM7. Evolutionary angles of the kink of TM7 with P327^7.50^ as the pivot point (**A**) and the distribution profile of these angles (**B**) among our three 600 ns systems.

A sharp kink on TM7 was observed in the crystal structure of the *κ*OR^26^ with P327^7.50^ as the pivot point^36^,^37^. Although this kink was preserved in our simulations, the kink angles behave differently in the three systems. Temporal evolutions of the kink angle of TM7 indicate that the kink in the 5′-GNTI-*κ*OR system is more stable than that in the other two systems. The kink angle is stabilized at about 150^o^ in the 5′-GNTI-*κ*OR complex (Fig. 7 and Table S1). However, the kink angle in the 6′-GNTI-*κ*OR complex or in the apo form shows an obvious deviation. Specifically, this kink angle in the 6′-GNTI complex changed from about 150^o^ to 130^o^ at the initial stage of the simulation. The temporal evolution of the kink angles could be used to explain the responses of TM7 caused by GNTIs.

A detailed analysis of the simulation results reveals that the kink is stabilized by a hydrogen bond formed between S324^7.47^ and V69^1.42^ (Table 2). The occupancy rate of this hydrogen bond is 24% from the simulation of the 5′-GNTI-*κ*OR system, which is much higher than that in the 6′-GNTI-*κ*OR system (2.7%) and in the apo *κ*OR (6.1%). A recent study on CRF_1_R also proved the significance of the kink in explaining the antagonistic mechanism^41^.

### Allosteric action mediated by the sodium ion

The role of the sodium ion in the function of some GPCRs has become an attractive topic recently. The crystallographic structure of the adenosine A2A receptor (A2AR) at an ultra-high resolution provides us with the first evidence of the binding of the sodium ion to the GPCR, followed by the crystallographic structures of the *β*1-adrenergic receptor and *δ*OR^28^,^42^,^43^. In the *δ*OR, the sodium ion is positioned around the negatively changed D^2.50^, and coordinated by two polar residues (N131^3.35^ and S135^3.39^) and two water molecules^28^. Many studies have been carried out to investigate the allosteric activation of GPCRs mediated by the sodium ion^44^. A recent molecular dynamics simulation of the *μ*OR suggested that a sodium ion penetrates into the allosteric pocket from the extracellular side of the receptor to perform its allosteric effects on the receptor^40^. The dynamic properties of the sodium ion in the apo-*κ*OR have also been described in the latest work of Filizola *et al.*^45^.

**Figure 8:**
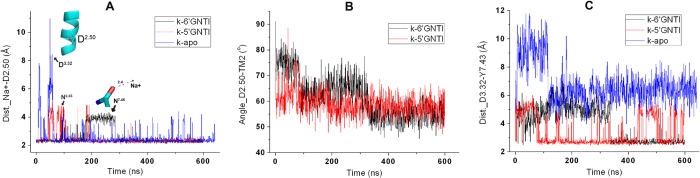
The evolutions of D105^2.50^ and 3-7 lock. (**A,B**) Evolution of the distance between Na+ and the conserved residue D105^2.50^ (**A**) or the angle between D105^2.50^ and TM2 (**B**). (**C**) Variation of the distance between the residue D138^3.32^ and Y320^7.53^.

In the crystal structure of the *κ*OR, the sodium ion is not co-crystallized with the *κ*OR. We thus placed a sodium ion in the allosteric pocket of the *κ*OR by referring to the position of the co-crystallized sodium ion in the crystal structure of the NTI-*δ*OR complex^28^. In our simulations, the sodium ion is stablized in the allosteric pocket, reflecting that the position of the sodium ion is reasonable (Fig. 8A).

In our simulations, although the sodium ion is stablized in the allosteric pocket, the residues coordinating the sodium ion undergo conformational changes and result in the fluctuation of the distances between the sodium ion and the residues (Fig. 8A). For example, the distance between the sodium ion and D105^2.50^ increased significantly during 180–300 ns in the simulation of the 6′-GNTI-*κ*OR system due to the conformational change of D105^2.50^ (Fig. 8B); D105^2.50^ deviated from the allosteric pocket and N322^7.45^ was involved in the direct interaction with the sodium ion during 180–280 ns.

### The 3-7 lock

Formation of a hydrogen bond between D138^3.32^ and Y320^7.43^, which is named as the 3–7 lock in the following text, is suggested to play a key role in the activation of the *κ*OR. To investigate the 3–7 lock in detail, we monitored the temporal evolution of the distance between D138^3.32^ and Y320^7.43^ (Fig. 8C). In the 5′-GNTI-*κ*OR system, a hydrogen bond between D138^3.32^ and Y320^7.43^ formed at about 80 ns and broke at about 500 ns of the simulation. The occupation rate of the hydrogen bond was 69.6%. In the simulation of the 6′-GNTI-*κ*OR system, a hydrogen bond between D138^3.32^ and Y320^7.43^ formed at about 340 ns and was preserved until the end of the simulation, resulting in an occupation rate of 44.4%. On the contrary, the 3–7 lock was not found in the whole simulation of the *κ*OR in the apo form. We thus contribute the formation of the hydrogen bond between D138^3.32^ and Y320^7.43^ to the binding of 5′-GNTI or 6′-GNTI to the orthosteric pocket of the *κ*OR.

### The antagonistic effects of 5′-GNTI

**Figure 9:**
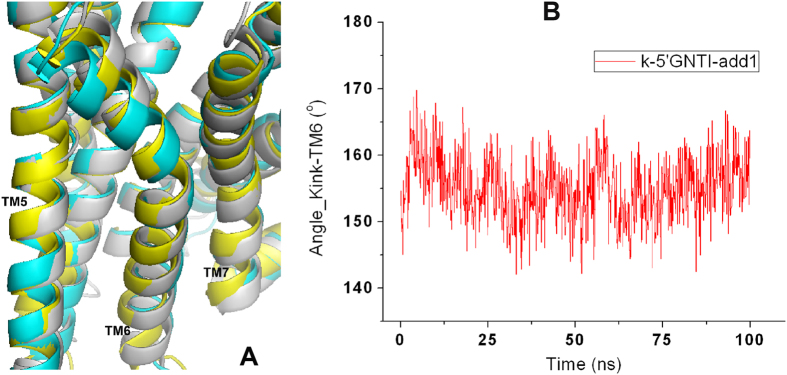
The conformational changes of TM6 in additional 5′-GNTI system. (**A**) Structural alignment of *κ*ORs with initial crystal structure (gray), principal component of additional 5′-GNTI-*κ*OR complex (yellow) and 600 ns state of 6′-GNTI-*κ*OR complex (blue). (**B**) Evolution of the kink angle of TM6 in additional 100 ns 5′-GNTI system.

To investigate whether 5′-GNTI can trigger a change of the conformation of the *κ*OR induced by 6′-GNTI to the inactive conformation, we docked 5′-GNTI to the last snapshot the *κ*OR generated in the simulation of the 6′-GNTI-*κ*OR system (Fig. S6), and performed another MD simulation with a time scale of 100 ns. Interestingly, we found that TM6 gradually returned to the antagonistic state under the action of 5′-GNTI (Fig. 9A) as indicated by the kink angle of TM6. This angle changed from about 160^o^ to 145^o^ at the beginning of the simulation, and then increased to 157 ± 4^o^ (Fig. 9B and Table S1). Structural alignments of TM6 also indicated that 5′-GNTI triggered the conformational change of the *κ*OR induced by 6′-GNTI to the inactive state gradually. In addition, the tilt direction of the indole ring was outward in Cluster 1 of the additional 5′-GNTI, which was identical with the 600 ns 5′-GNTI-*κ*OR system (Table 1 and Fig. S2).

## Discussion

In our simulations, we found that E297^6.58^ in the *κ*OR is directly interacting with 6′-GNTI. This interaction, together with the steric effect from I294^6.55^, contributes to the rotation of TM6 as well as the movements of other TMs. In contrast, this ligand shows no agonistic effect towards the *μ*OR and *δ*OR^23^. From sequence alignments of the *κ*OR, *μ*OR and *δ*OR, we can easily find that E^6.58^ in the *κ*OR corresponds to K^6.58^ in the *μ*OR and to W^6.58^ in the *δ*OR^46^. On the other hand, it has been suggested that both K^6.58^ in the *μ*OR and W^6.58^ in the *δ*OR are not directly interacting with the guanidinium group of 6′-GNTI, which leads to the absence of the agonistic effect of this ligand towards the *μ*OR and *δ*OR^23^. All these observations demonstrate that the direct interaction between the residue E297^6.58^ and 6′-GNTI plays an indispensible role in the activation of the *κ*OR and the residues E297^6.58^ and I294^6.55^ function as a critical pair in the activation of the *κ*OR.

Besides the nontrivial function of E297^6.58^ and I294^6.55^ in the activation of the *κ*OR, large conformation changes of TM7 and TM2 were also identified in our simulations as indicated by the kink angles of TM7 with P327^7.50^ as the pivot point (Fig. 7) and TM2 with D105^2.50^ as the pivot point (Fig. 8B). Such conformational changes can further help us understand the mechanism of *κ*OR activation. In addition, D105^2.50^ is the key residue in positioning the sodium ion in the allosteric pocket. Therefore, we believe that the sodium ion also contributes to the activation of the *κ*OR.

S324^7.47^ is located adjacent to the pivot point P327^7.50^ on TM7 and forms a hydrogen bond with a residue on TM1 in the simulation of the 5′-GNTI-*κ*OR system (Fig. 7 and Table 2). Because of the formation of the hydrogen bond, the conformational change of TM7 is hampered. As a result, TM7 is stabilized in its initial conformation and leads to that the *κ*OR adopts the inactive conformation. We thus suggest S324^7.47^ plays an important role in stabilizing the *κ*OR at the inactive state.

The 3-7 lock is found to be formed in our simulations of the 5′-GNTI- *κ*OR and 6′-GNTI-*κ*OR systems. In contrast, such a lock is breaking-down in the crystal structure of the JDTic-*κ*OR complex. Both 5′-GNTI and 6′-GNTI are derivatives of morphinan, while the ligand JDTic^47^ is a pure antagonist with (3R,4R)-3,4-dimethyl-4-(3-hydroxyphenyl)piperidine as the scaffold. We can thus use the ligand in the orthosteric pocket to explain the formation and breaking-down of the 3-7 lock in the *κ*OR - the derivatives of morphinan can induce the formation of the 3-7 lock, while a pure antagonist results in the breaking-down of such a lock in the *κ*OR.

In addition, we observed a large rotation of TM6 around the pivot point P289^6.50^ in the simulation of the 6′-GNTI-*κ*OR system, which is an indispensible step for the activation of the *κ*OR. In our opinion, the receptor needs to visit a set of intermediate states for its activation from the inactive state to the fully active state^48^. The rotation of TM6 allows the receptor to visit one of the key intermediate states for the activation of the *κ*OR. However, we did not observe full activation of the receptor probably due to the limitation of the available computational resources.

## Conclusions

In this work, we studied the molecular switches of the *κ*OR triggered by 5′-GNTI and 6′-GNTI using molecular dynamics simulations. We observed about 4.6^o^ rotation of TM6 on average in the *κ*OR-6′-GNTI complex. Detailed analysis of the simulation results revealed that E297^6.58^ and I294^6.55^ play a crucial role for the rotation of TM6. On the other hand, we found that the hydrogen bond between S324^7.47^ and residue V69^1.42^ on TM1 contributes to the stabilization of the *κ*OR in the inactive state as revealed from the simulation of the *κ*OR-5′-GNTI complex.

## Materials and Methods

### Protein preparations

The published crystal structure of human inactive JDTic-bound *κ*OR (PDB code: 4DJH), was obtained from an engineered *κ*OR mutant protein where part of the intracellular loop between transmembrane helices TM5 and TM6, was replaced by T4 lysozyme (T4L)^26^. In order to do molecular dynamics simulations on the wild type receptor based on the crystal structure of the T4L mutant *κ*OR dimer, we removed the T4L from the mutant receptor structure, the monomer B and other unnecessary parts. Then we reconstructed the loop of the remaining chain A by adding the missing residues S262 and T302–S303–H304–S305–T306 using the loop refinement protocol in Discovery Studio 3.5; 10 loops were generated and the most reasonable one was chosen for receptor construction. According to the latest crystal structure of inactive NTI-complexed δOR, ion Na+ was exactly located at the allosteric pocket position^28^.

The above three-dimensional integrated *κ*OR structure was then imported into the Schrödinger software package. The protein structure was prepared with Protein Preparation Tool (ProPrep) in the Schrodinger 2012 suit software. Asn, Gln, and His residues checked for protonated states automatically in ProPrep. Hydrogen atoms were added into the *κ*OR crystal structure at the physiology pH environment by the PROPKA tool in Maestro with optimized hydrogen-bond network. No non-standard protonation state of the amino acids was found.

### Molecular docking

By referring to the conformation of ligand NTI in crystal structures of *δ*OR, *κ*OR-selective antagonist 5′-GNTI and *κ*OR-selective agonist 6′-GNTI were sketched in Maestro and subjected to a Monte Carlo Μultiple Minimum conformational search using the OPLS_2005 force field and water as implicit solvent (Surface Generalized Born (SGB) model). The output conformations were used as the starting point for the docking experiments.

The pocket Grid file of the *κ*OR was generated by 20 Å around residue D138^3.32^ with the Receptor Grid Generation module, and docked with Glide Docking module (Glide 5.8)^49^,^50^. The Van der Waals (vdW) scaling was set to 0.8 for nonpolar atoms of receptor and ligand. During docking, the number of docking output was set as 10000 poses per docking run at most. The most reasonable conformation was picked out for molecular dynamics simulations.

### MD preparations

Two 5′-GNTI-*κ*OR complexes, one 6′-GNTI-*κ*OR complex and one apo-*κ*OR system, were built for the simulations. A POPC (1-palmitoyl-2-oleoyl-sn-glycero-3-phosphocholine) bilayer with the surface area of 75 × 75 Å^2^ on the X-Y plane was generated under Charmm36 force field by VMD program (Version 1.9.1). For each system, the receptor was first embedded into the POPC bilayer using our in-house program pre-aligned in the OPM (Orientations of Proteins in Membranes) database^51^,^52^,^53^,^54^. Thereafter, Pre-equilibrated 103 POPC lipids coupled with 11067 TIP3P water molecules in a box ~75 × 75 × 100 Å^3^ were used solvate the protein. Lipid molecules within 0.85 Å of the heavy atoms on the protein structure and water molecules in the bilayer were removed. 51 Na+ and 59 Cl- ions were used to produce neutral systems with 0.15 M NaCl in the water phase of GNTI systems, whereas the number of Cl- ions was 57 in apo-*κ*OR system. We described the protein using CHARMM36 force field with cmap correction.

Ligand force field was generated by Paramchem webserver, a program coupled with CHARMM Force field^55^,^56^,^57^. Small molecules with a correct configuration were imported into Paramchem webserver and force field parameters of these compounds were then obtained through a script CHARMM General Force Field (CGenFF).

### Molecular dynamics simulations

All simulations were performed using Gromacs V.4.6.5^58^ and the CHARMM36 parameters for all compositions. In the first simulation step, the system was subjected to a 10000-step energy minimization with 1000.0 kJ/mol/nm as the force threshold. Then, the systems were gradually heated from 0 K to 310 K followed by 50 ps initial equilibration at constant volume and temperature at 310 K (NVT). An additional 1 ns equilibration was performed at constant pressure and temperature (NPT ensemble; 310 K, 1 bar) with two thermostats (stabilizing temperature independently for protein-ligand system, and the lipids-water-ions system) at 0032 ps time steps. vdW and short-range electrostatic interactions were cut off at 12 Å. Long-range electrostatic interactions were computed by the Particle Mesh Ewald (PME) summation scheme. The MD simulations of the additional 5′-GNTI system was performed for 100 ns, while all the other systems were performed for about 600 ns under NPT conditions with Parrinello-Rahman pressure coupler methods and Nose-Hoover thermostat for temperature coupling. The time step for MD simulation was 2 fs and the integrator leap-frog algorithm was employed. While the MD simulations of the additional 5′-GNTI-*κ*OR system was performed for 100 ns, all the other systems were performed for about 600 ns using the NPT ensemble with the Parrinello-Rahman pressure coupling and the Nose-Hoover temperature coupling methods.

### Analysis of simulations

RMSD and RMSF calculations, ligand cluster statistics, hydrogen bond analysis, angle and distance evolutions were produced by the program Gromacs. The interval time of trajectory calculations, including RMSD, RMSF, angle, distance and principal component analysis, was 500 ps in 600 ns systems, whereas that in 100 ns 5′-GNTI systems was 100 ps. RMSD values were calculated through comparing to the initial simulation conformations. Principal components analysis was carried on through the g_covar tool. All the smooth curves in Figs 4, 7 and Fig. S3 were fitting groups, which were identical with corresponding Angle/RMSD calculations in color.

To determine the sub-state conformations that close ligands bound to *κ*OR, structural clustering was performed using the g_cluster tool in Gromacs with the linkage algorithm based on the RMSD of each ligand molecule after alignment of the Cα atoms in the transmembrane helices in each frame to the starting structure^59^. A 0.2 Å RMSD cut-off was chosen because it best captured spatially distinct clusters and allowed the top clusters to be representative of the predominant binding sites explored. The clustering was performed on the simulation frames with 100 ps interval time. The populations of each cluster are given in Table 1. We examined the two most populated clusters and calculated the RMSDs for each ligand found in these clusters (Table 1).

Angle analysis was carried on by g_angle tool in Gromacs. The specific input parameters were as follows. The kink angle of TM6 was measured with atom C_α_ of V285^6.46^, P289^6.50^ and F293^6.54^; The angle between I294^6.55^ and TM6 was measured with atom C_δ_ and C_α_ of I294^6.55^ and atom C_α_ of W287^6.48^, while that angle between E297^6.58^ and TM6 was computed with atom C_δ_ and C_α_ of E297^6.58^ and atom C_α_ of F293^6.54^; The kink angle of TM7 was measured with atom C_α_ of Y320^7.43^, P327^7.50^ and A331^7.54^; The angle between D105^2.50^ and TM2 was measured with atom C_γ_ and C_α_ of D105^2.50^ and atom C_α_ of I98^2.43^.

NMA was conducted using the ElNemo^60^ (http://www.igs.cnrs-mrs.fr/elnemo/index.html), a web interface to the elastic network model-based NMA.

## Additional Information

**How to cite this article**: Cheng, J. *et al.* Molecular switches of the κ opioid receptor triggered by 6′-GNTI and 5′-GNTI. *Sci. Rep.* **6**, 18913; doi: 10.1038/srep18913 (2016).

## Supplementary Material

Supplementary Video 1

Supplementary Video 2

Supplementary Information

## Figures and Tables

**Figure 1:**
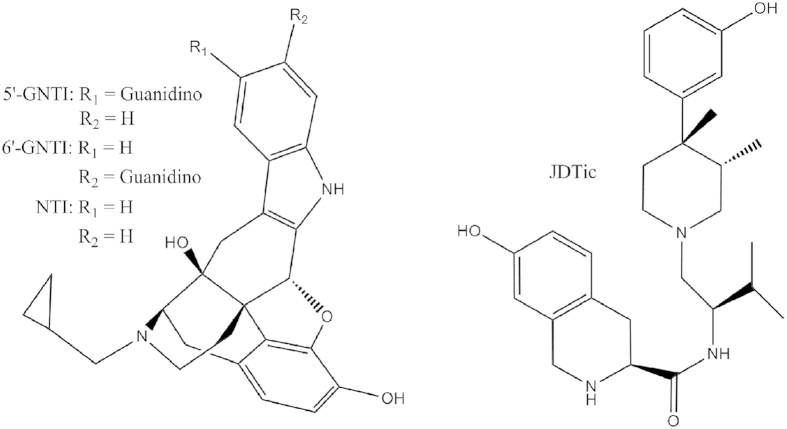
Two-dimensional structure formula of the agonist 6′-GNTI, antagonist 5′-GNTI, Naltrindole and JDTic.

**Table 1:** Percentage and average RMSD in angstroms of 5′-GNTI and 6′-GNTI that compose the clusters.

	5′-GNTI (600 ns)		6′-GNTI		5′-GNTI (additional)	
	Percentage	RMSD	Percentage	RMSD	Percentage	RMSD
Cluster 1	81.4	0.74	50.4	1.03	73.2	0.59
Cluster 2	10.7	0.54	45.4	0.83	19.4	0.43
Total	92.1		95.8		92.6	

**Table 2:** Occupancies of the hydrogen bonds in the two 600 ns GNTIs-*κ*OR systems and the apo-*κ*OR system.

HB NO.	5′-GNTI system			6′-GNTI system			Apo system	
	1(%)	2(%)	3(%)	1(%)	2(%)	3(%)	1(%)	2(%)
E6.58-ligand	0.4	93.9	5.7	2.7	89.5	7.3	—a	—
D3.32-ligand	71.4	2.3	—	19.1	6.6	1.1	—	—
S7.47-V1.42	24.3	—	—	2.7	—	—	6.1	—
D3.32-Y7.43	69.4	0.2	—	44.1	0.3	—	—	—

^a^the symbol “–” represents that there is no hydrogen bond interaction.
