# Supplementary material for: Molecular switches of the κ opioid receptor triggered by 6′-GNTI and 5′-GNTI
Source: Sci Rep. 2016 Jan 8;6:18913. doi: 10.1038/srep18913 (PMC4705513; doi:10.1038/srep18913)
Supplement: Supplementary Information [file srep18913-s3.doc]

# Support information

# Molecular switches of the *κ* opioid receptor triggered by 6’-GNTI and 5’-GNTI

Jianxin Cheng^a^, Xianqiang Sun^b^, Weihua Li^a^, Guixia Liu^a^, Yaoquan Tu*^,b^, Yun Tang*^,a^

^a^ Shanghai Key Laboratory of New Drug Design, School of Pharmacy, East China University of Sc**i**ence and Technology, Shanghai 200237, China

^b^ Division of Theoretical Chemistry and Biology, School of Biotechnology, KTH Royal Institute of Technology, S-106 91 Stockholm, Sweden

Corresponding authors:

*, a Email: [ytang234@ecust.edu.cn](mailto:ytang234@ecust.edu.cn) (Y. Tang)

*, b E-mail: [tu@theochem.kth.se](mailto:tu@theochem.kth.se) (Y. Tu)

**Article type:** Full-length paper

**Support information**

|  | 5’-GNTI system  (600 ns) | 6’-GNTI system  (600 ns) | 6’-GNTI system  (last 300 ns) | Apo system  (600 ns) | additional 5’-GNTI system (last 10 ns) |
| --- | --- | --- | --- | --- | --- |
| Angle of the kink of TM6 | 160 ± 4 | 156 ± 6 | 159 ± 4 | 159 ± 4 | 157 ± 4 |
| Angle between I294^6.55^ and TM6 | 81 ± 7 | 63 ± 14 | -- | 61 ± 16 | -- |
| Angle between E294^6.58^ and TM6 | 86 ± 7 | 77 ± 9 | -- | -- |  |
| Angle of the kink of TM7 | 150 ± 3 | 146 ± 5 | 150 ± 3 | 150 ± 4 | -- |
| Minimum distance between C_α_ of I294^6.55^ and GNTIs | 5.5 ± 0.2 | 5.2 ± 0.3 | -- | -- | -- |
| RMSD of TM3 | 1.3 ± 0.2 | 1.3 ± 0.3 | -- | 1.0 ± 0.1 | -- |

**Table. S1** Average values of angles in degree (^o^), minimum distance and RMSD in angstroms (± standard deviation) among our simulations.

**Support information**

**Fig. S1** RMSD evolution of ligands in 600 ns GNTIs-*κ*OR systems during our simulations.

**Fig. S2** Representative poses of ligand clusters 1 and 2 and relative tilt direction of indole ring at the extracellular side in 6’-GNTI-*κ*OR complexes (A), 600 ns 5’-GNTI-*κ*OR systems (B) and additional 5’-GNTI-*κ*OR systems (C). Color green and blue respectively mean the frames from clusters 1 and 2.

**Fig. S3** Distribution profiles of the angles (kink of TM6) (A) and RMSD of TM3 (B) among our three 600 ns systems.

**Fig.** **S4** Movement directions (arrows) of TM helices at the extracellular side in 600 ns 5’-GNTI-*κ*OR complexes (A) and apo-*κ*OR systems (B) during dynamics simulations. Color gray, green and blue respectively represent the initial structure, principal component and 600 ns frame.

**Fig. S5** Evolution of the angles between residue I294^6.55^ and TM6 (A) or between residue D105^2.50^ and TM2 (B) in apo-*κ*OR systems.

**Fig. S6** Docking pose of 5’-GNTI (orange) with the protein structure extracted from the 600 ns 6’-GNTI-*κ*OR complex. Hydrogen bond interactions were shown in yellow dot lines.


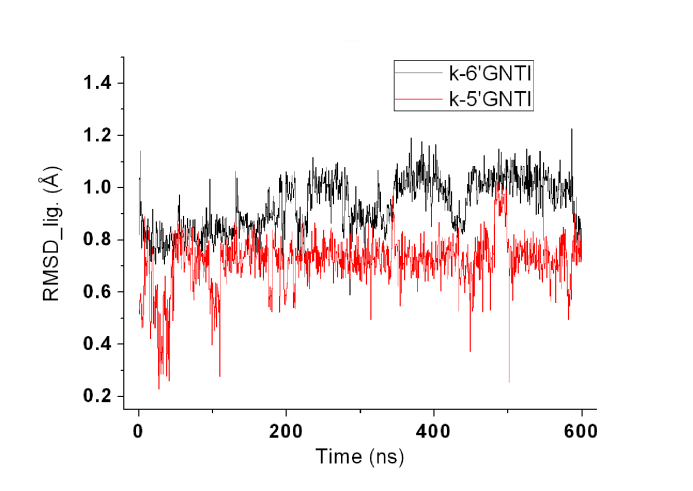


**Fig. S1** RMSD evolution of ligands in 600 ns GNTIs-*κ*OR systems during our simulations.


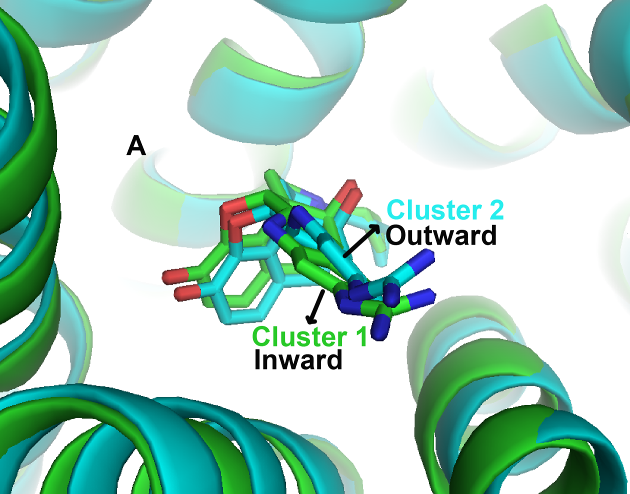


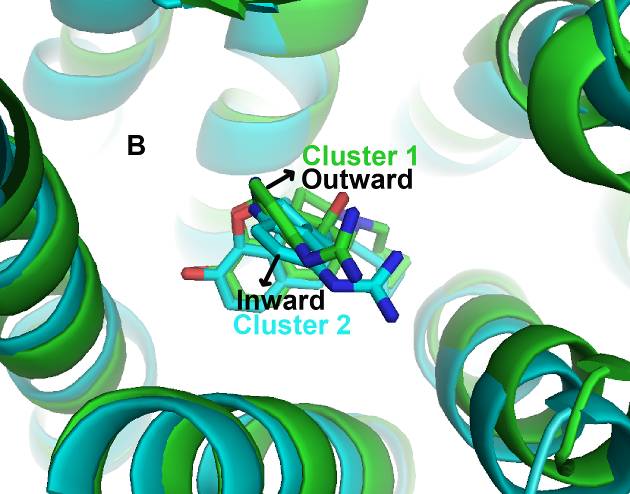


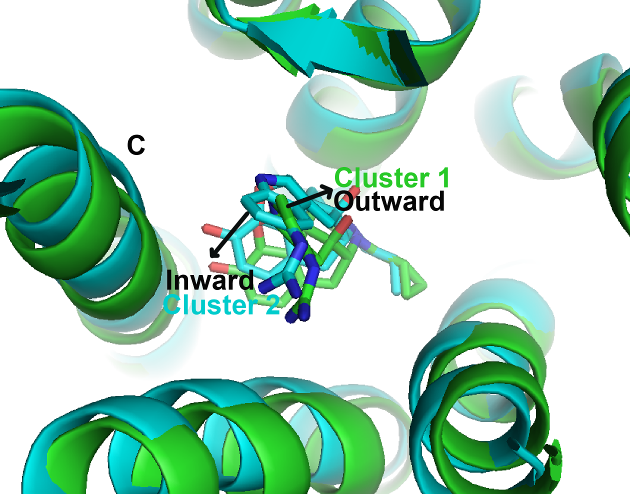


**Fig. S2** Representative poses of ligand clusters 1 and 2 and relative tilt direction of indole ring at the extracellular side in 6’-GNTI-*κ*OR complexes (A), 600 ns 5’-GNTI-*κ*OR systems (B) and additional 5’-GNTI-*κ*OR systems (C). Color green and blue respectively mean the frames from clusters 1 and 2.


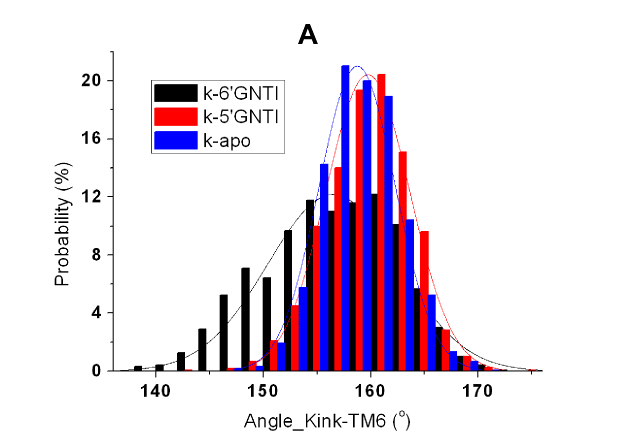


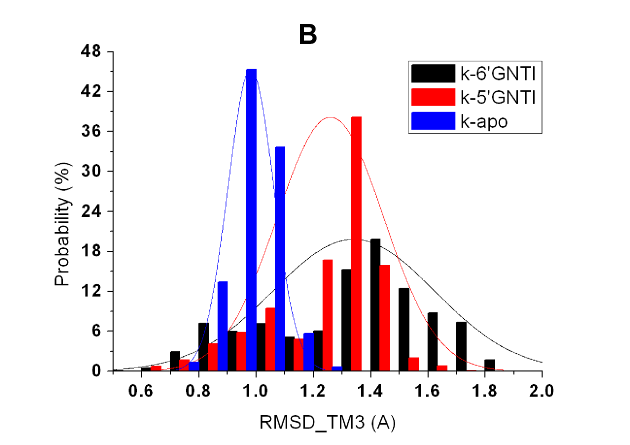


**Fig. S3** Distribution profiles of the angles (kink of TM6) and RMSD of TM3 among our three 600 ns systems.


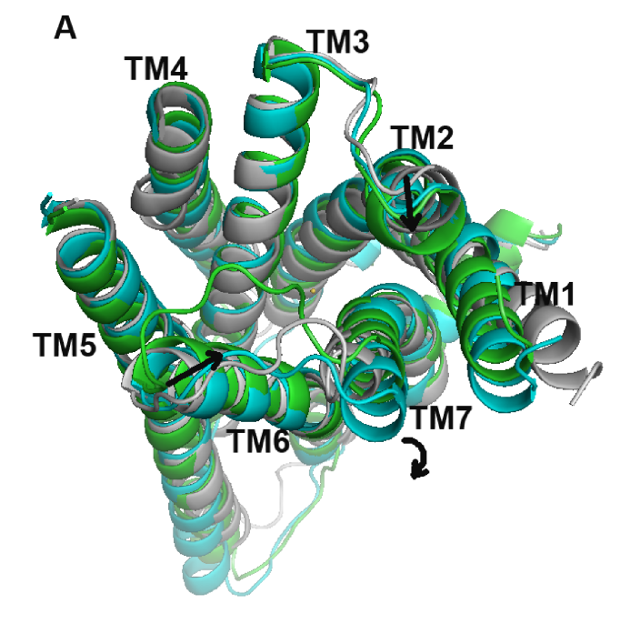


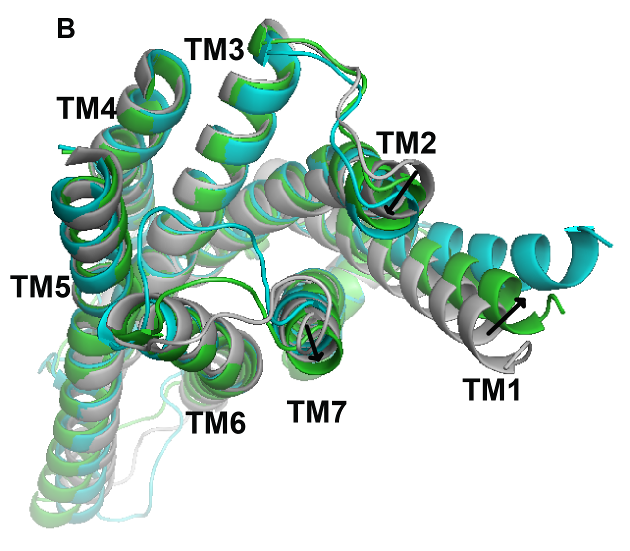


**Fig. S4** Movement directions (arrows) of TM helices at the extracellular side in 600 ns 5’-GNTI-*κ*OR complexes (A) and apo-*κ*OR systems (B) during dynamics simulations. Color gray, green and blue respectively represent the initial structure, principal component and 600 ns frame.

**
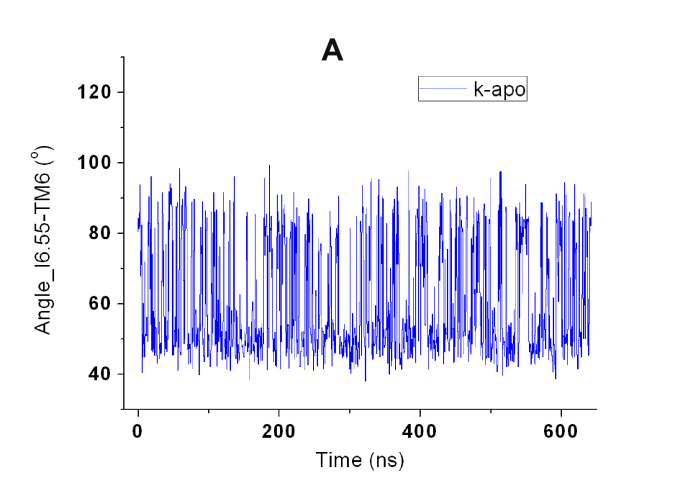
**

**
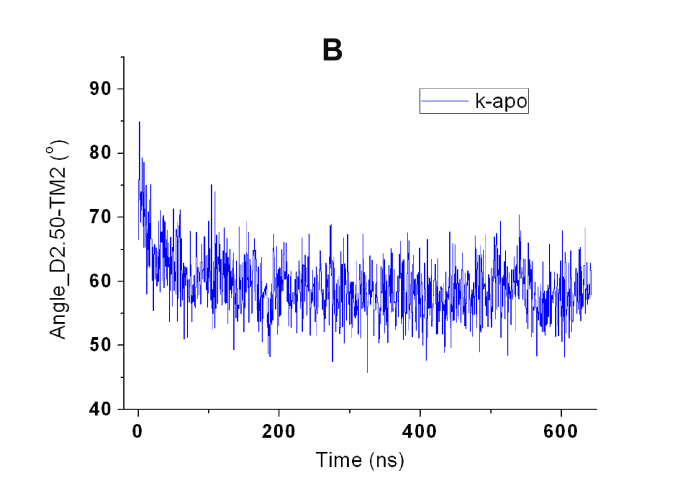
**

**Fig. S5** Evolution of the angles between residue I294^6.55^ and TM6 (A) or between residue D105^2.50^ and TM2 (B) in apo-*κ*OR systems.


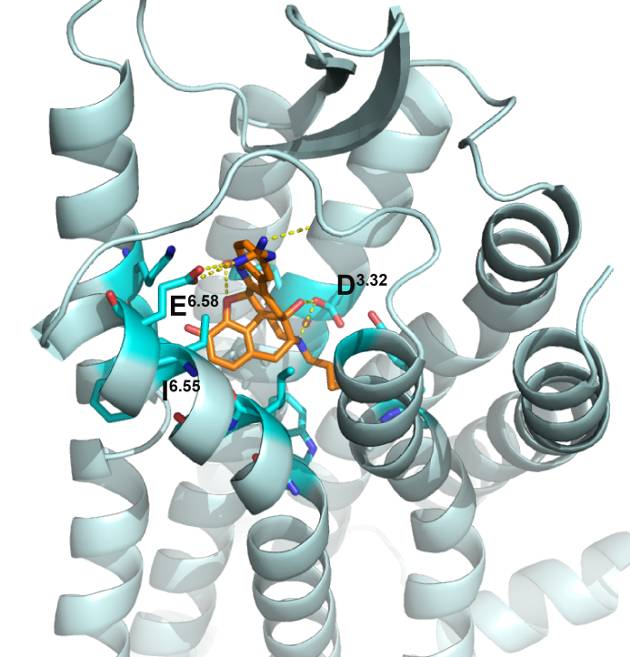


**Fig. S6** Docking pose of 5’-GNTI (orange) with the protein structure extracted from the 600 ns 6’-GNTI-*κ*OR complex. Hydrogen bond interactions were shown in yellow dot lines.

**Support information**

**Video S1** The evolution overview of 5’-GNTI-*κ*OR systems (A) or 6’-GNTI-*κ*OR systems (B) during our 600 ns simulations.
